# Possible role of plasma Galectin-9 levels as a surrogate marker of viremia in HIV infected patients on antiretroviral therapy in resource-limited settings

**DOI:** 10.1186/s12981-020-00298-9

**Published:** 2020-07-16

**Authors:** Ashwini Shete, Sampada Dhayarkar, Ashwini Dhamanage, Smita Kulkarni, Manisha Ghate, Shashikala Sangle, Uttam Medhe, Vinita Verma, Shobini Rajan, Toshio Hattori, Raman Gangakhedkar

**Affiliations:** 1 ICMR-National AIDS Research Institute, 73-G Block, M.I.D.C, Bhosari, Pune, India; 2 B.J. Medical College and Sassoon General Hospital, Jai Prakash Narayan Road, Near Pune Railway Station, Pune, India; 3 Yashwantrao Chavan Memorial Hospital, Sant Tukaram Nagar, Pimpri, Pune, India; 4 National AIDS Control Organization, Chandralok Building, 36, Janpath, New Delhi, India; 5 Kibi International University, Takahashi, Okayama Japan; 6 Present Address: National Centre for Cell Sciences, University of Pune Campus, University Road, Ganeshkhind, Pune, India; 7 Present Address: Indian Council of Medical Research, V. Ramalingaswami Bhawan, Ansari Nagar, P.O. Box No. 4911, New Delhi, India

**Keywords:** HIV, Galectin-9, Surrogate marker, Viral load, Immune-activation markers

## Abstract

**Background:**

Early detection of viremia in HIV infected patients on anti-retroviral therapy (ART) is important to prevent disease progression as well as accumulation of drug resistance mutations. This makes HIV viral load (VL) monitoring indispensable in HIV infected patients on ART. However VL, being an expensive test, results in heavy financial burden on health services. Hence, cheaper surrogate markers of viremia are desired to reduce overall cost of management of HIV infected patients.

**Methods:**

We enrolled aviremic (n = 63, M:F = 31:32) and viremic (n = 43, M:F = 21:22) HIV infected patients at 1 year after ART initiation. Viremic individuals were identified as those having a plasma VL of more than 1000 copies/µl and aviremic individuals as less than 40 copies/µl. The study participants also included immuno-virologically discordant patients as they demonstrate differential degrees of immune-reconstitution and are likely to harbour concomitant infections influencing levels of immune-activation markers screened as the surrogate markers. Immune activation markers viz. plasma hs-CRP, soluble-CD14 and Galectin-9 levels were estimated by ELISA, IL-6 by luminex assay and percentages of CD38+ CD8+ cells were determined by flow cytometry. The levels were compared between viremic and aviremic patients and correlated with plasma viral load. Receiver operated curve (ROC) analysis was done for plasma Galectin-9 levels.

**Results:**

Viremic patients had significantly higher levels of Galectin-9 and %CD38+ CD8+ cells (p values < 0.0001) than aviremic patients. Levels of the other activation markers did not differ between viremic and aviremic individuals. Galectin-9 levels (r = 0.76) and %CD38+ CD8+ cells (r = 0.39) correlated positively with VL. Area under curve for Galectin-9 levels for distinguishing between viremic and aviremic individuals was 0.98. Youden index, sensitivity, specificity, positive predictive value and negative predictive value for Galectin-9 levels were 0.87, 0.97, 0.90, 0.87 and 0.98, respectively, at the cut-off value of 5.79 ng/ml.

**Conclusions:**

Plasma Galectin-9 levels could identify viremic individuals with sensitivity and specificity of more than 90%. Thus, they showed a potential to serve as a surrogate marker of viremia in HIV infected patients on ART and would have cost implications on HIV management especially in resource-limited settings. However, the findings need to be confirmed in the patients on ART for different durations of time.

## Introduction

Antiretroviral therapy (ART) has significantly impacted Human Immunodeficiency Virus (HIV) epidemic worldwide. The primary goal of ART is to suppress HIV viral load. UNAIDS 90-90-90 target for elimination of HIV as a public health threat includes achievement of viral suppression in the treated individuals as its last ‘90’ [1]. Failure to achieve viral suppression after ART initiation increases the risk of disease progression in them. Continued viral replication in presence of drug pressure has been shown to lead to development of drug resistant (DR) mutations. There is also a risk of development of multiple DR mutations if viremia persists for long time [2] further compromising options for second-line therapy. Hence early detection of viremia is a key to successful management of HIV infected patients on ART. Moreover, unsuppressed viremia increases the risk of secondary transmission of HIV which might lead to spread of drug resistant strains in the community. Hence, timely detection of failure of viral suppression is utmost important to achieve sustained control of HIV epidemic.

WHO has recommended annual viral load testing for monitoring HIV infected patients on ART [3]. However, viral load estimation is an expensive test requiring a sophisticated equipment, costly kits and skilled manpower. Hence, the patients are still monitored using clinical assessment or CD4 counts estimation in resource-poor countries [4]. Different point of care tests (POCTs) for viral load monitoring are also being evaluated to overcome the challenges posed by viral load testing. However robust POCTs offered at an affordable price are still not available [5]. Among 36.7 million estimated people living with HIV/AIDS globally, a vast majority reside in low- and middle-income countries [6]. Cost of viral load testing creates a heavy financial burden on these patients as well as on the national programs for HIV control run in such countries. This cost is incurred every year as there is no cure for HIV at present. Hence, cheaper surrogate biomarkers are desired to cut down this cost especially in low- and middle-income countries.

There have been several studies to identify surrogate biomarkers of viremia. CD4 count is being used as a marker of treatment success and is a part of patient management under our programme also. However, immune-virologically discordant responses are known to occur in 8–24% of the patients on ART [7] reducing sensitivity and specificity of the test in detecting viremic individuals. Hemoglobin and total lymphocyte count have been studied and shown to be reliable predictors of successful treatment outcome comparable to the increase in CD4 count [8]. CD38 expression has also been shown to correlate with viremia and has been proposed as a surrogate biomarker [9, 10]. Many of circulating immune activation markers have also been studied and shown to distinguish viral suppression from nonsuppression in HAART-treated patients [11].

We evaluated immune activation markers like high sensitivity C-reactive protein (hs-CRP), soluble CD14 (sCD14), bacterial lipopolysaccharide (LPS), Interleukin-6 (IL-6), Galectin-9, CD38 expressing CD8+ cells to determine their role as a possible surrogate marker of viremia. Being immune activation markers, their elevated levels have been reported in different infections even in HIV uninfected individuals. However, these markers were also shown to correlate with HIV viral loads in different studies and hence were selected in the present study [10, 12–16]. Among all these markers, only Galectin-9 has been shown to induce HIV reactivation in resting CD4 cells [12] indicating its role in contributing to viremia influencing viral load values. Although LPS has been shown to induce HIV reactivation through TLR4, it did not induce HIV reactivation in resting CD4 cells in one of the previous studies [17, 18]. Apart from HIV viremia driven activation, co-existing infections are also important drivers of immune activation [19]. Extent of immune-reconstitution is also likely to influence the levels of these markers as they are secreted by the cells of immune-system. Immuno-virologically discordant responders are more susceptible to infectious diseases than the treatment responders and they also represent individuals with differing degrees of viremia as well as immune-reconstitution. Hence we considered including the immuno-virologically discordant responders in addition to the concordant treatment responders and failure patients to evaluate the surrogate markers of viremia irrespective of the presence co-existing infections as well as the extent of immune-reconstitution.

## Materials and methods

### Characteristics of the study participants

This was a cross-sectional study conducted at ICMR-National AIDS Research Institute (ICMR-NARI). HIV infected patients at 1 year after initiation of anti-retroviral therapy were enrolled from Yashwantrao Chavan Memorial Hospital (YCM) and B.J. Medical College (BJMC) ART centers. HIV infected patients visiting these centres were screened based on their CD4 counts at baseline and at 1 year as well as viral load values at 1 year to identify the eligible study participants. Patients with the rise of at least 100 CD4 cells/μl and viral load of less than 40 copies/ml were selected as treatment responders. Patients with the rise of less than 50 CD4 cells/μl and viral load of less than 40 copies/ml were selected as Immunologic non-responders (INR). Viremic patients with viral load of more than 1000 copies/ml were enrolled under two categories. Treatment failures had immunologic failure as defined under the national guidelines and Virologic non-responders (VNR) had an increase of at least 50 CD4 cells/μl. Blood samples collected after written informed consenting procedure. Plasma and PBMC were separated by density gradient centrifugation using Ficoll-Hypaque.

### ELISA

Plasma concentrations of Galectin-9, hs-CRP, sCD14 were measured using commercially available ELISA Kits (R&D Systems, USA, and Biocheck Inc., USA). The ELISAs were performed according to manufacturers’ manuals. Concentrations of the immune-activation markers in the samples were determined by plotting standard curve as per the manufacturer’s instructions.

### Endotoxin assay

Plasma LPS levels were determined using Limulus Amebocyte assay (GenScript Biotech Corp., USA). Plasma samples were heat-inactivated by incubating at 60 °C for 30 min before measuring the levels.

### Flow cytometry

Frequency of CD38+ CD8+ cells in the study participants was determined in by flow cytometry using frozen PBMCs. PBMCs were revived and rested for 2 h before staining them with anti-CD3 PE/Dazzle 594 (Biolegend, USA), anti-CD8 APC/Cy7 and anti-CD38 FITC (both from BD Biosciences, USA) as described previously [20]. The cells were analysed on FACSAria Fusion using FACSDiva software (BD Biosciences, USA).

### Luminex assay

Interleukins 6 (IL-6) levels were estimated in plasma samples by a luminex assay along with other proinflammatory cytokines using Bio-Plex 200 system (Bio-Rad, USA) as per the manufacturer’s instructions.

### Data analysis

Data analysis was done using GraphPad Prism software. Non-parametric tests were used for the statistical analysis. Mann–Whitney (one-tailed analysis) test was used for comparison between viremic and aviremic groups. Multiple group comparisons were assessed through Kruskal–Wallis test with Dunn’s multiple comparison testing. Correlations with viral loads and CD4 counts were done using Spearman correlation test. easyROC: a web-tool (ver. 1.3.1) was used for receiver operating characteristic (ROC) curve analysis.

## Results

Characteristics of patients enrolled in the study are mentioned in Table 1. A total of 63 aviremic patients (Male:female 31:32) with age range of 23–62 years were enrolled in the study. Out of them 40 were responders and 23 were immunologic non-responders. Their enrolment median CD4 counts were 479 (range: 246–1387) cells/cmm and 227 (range: 13–360) cells/cmm, respectively. Viremic patients (n = 43; Male:female 21:22) were either treatment failure (n = 18) or virologic non-responders (n = 25). Their age ranged from 18 to 55 years. Their enrolment median CD4 counts were 89 (range: 19–331) cells/cmm and 317 (range: 99–809) cells/cmm, respectively. Their viral load varied from 1891 to 526,175 (median: 63,165) and 1056-889079 (median: 12,366) copies/ml, respectively.

**Table 1:** Characteristics of the participants enrolled in the study

Groups median (range)	Immunologic non responders (n = 23)	Matched responders (n = 40)	Treatment failure (n = 18)	Virologic non responders (n = 25)	P value viremic vs aviremic
Age: years	42 (23–60)	39.5 (25–62)	37.5 (24–52)	35 (18–55)	0.016
Baseline CD4: cells/cmm	270 (14–380)	283.5 (25–352)	142.5 (8–358)	159 (22–350)	< 0.0001
Enrolment CD4: cells/cmm	227 (13–360)	479 (246–1387)	89 (19–331)	317 (99–809)	< 0.0001
Viral load (copies/ml)	< 40	< 40	63,165 (1891–526,175)	12,366 (1056–889,079)	< 0.0001

**Fig. 1:**
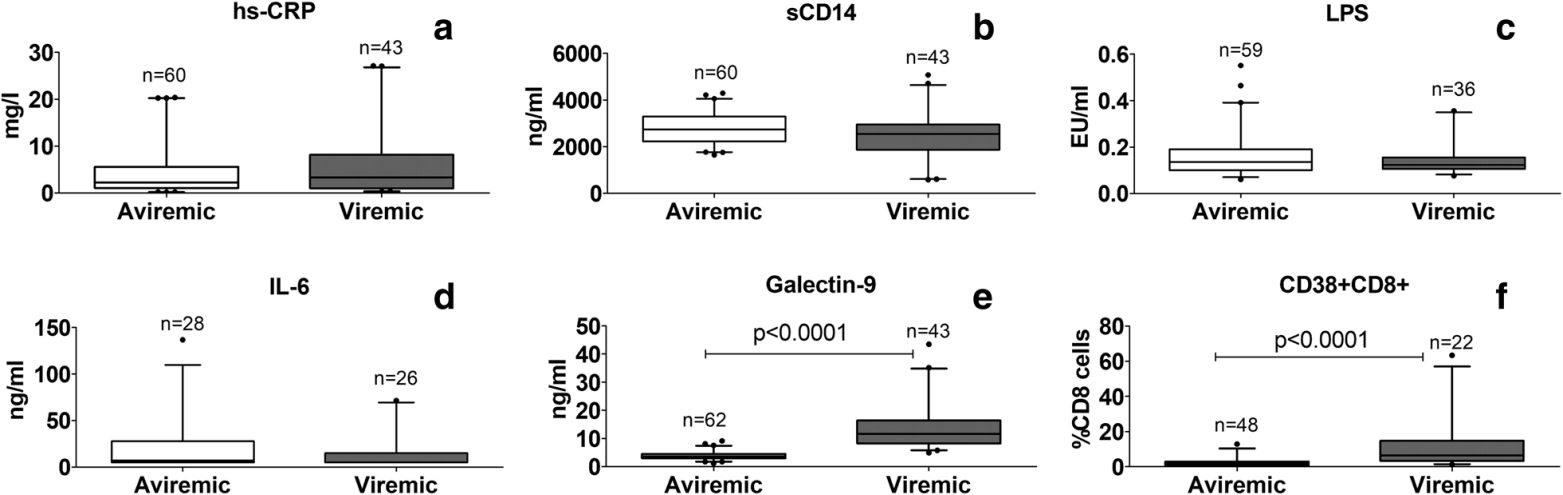
Comparison of immune-activation markers in viremic and aviremic HIV infected patients. The figure shows levels of **a** hs-CRP (mg/l), **b** sCD14 (ng/ml), **c** LPS (EU/ml), **d** IL-6 (ng/ml), **e** Galectin-9 (ng/ml), and **f** frequency of CD38+ CD8+ cells plotted on Y axis. Medians values and interquartile ranges for the groups are plotted as bars and error bars. Aviremic group is indicated as open bar and viremic group is indicated by black coloured bars. Number of samples used for the analysis are mentioned above the bars. P values calculated by Mann–Whitney test showing significant difference between the groups as are shown in the figure

Systemic immune activation was assessed by estimating CD38 expressing CD8+ cells and soluble markers like hs-CRP, sCD14, LPS, IL-6 and Galectin-9. Levels of these markers were compared between viremic versus aviremic individuals (Fig. 1). Levels of hs-CRP, sCD14, LPS, IL-6 did not vary significantly between the viremic and aviremic individuals. However, plasma Galectin-9 levels and frequency of CD38 expressing CD8+ cells differed significantly among these two groups (p < 0.0001).

**Fig. 2:**
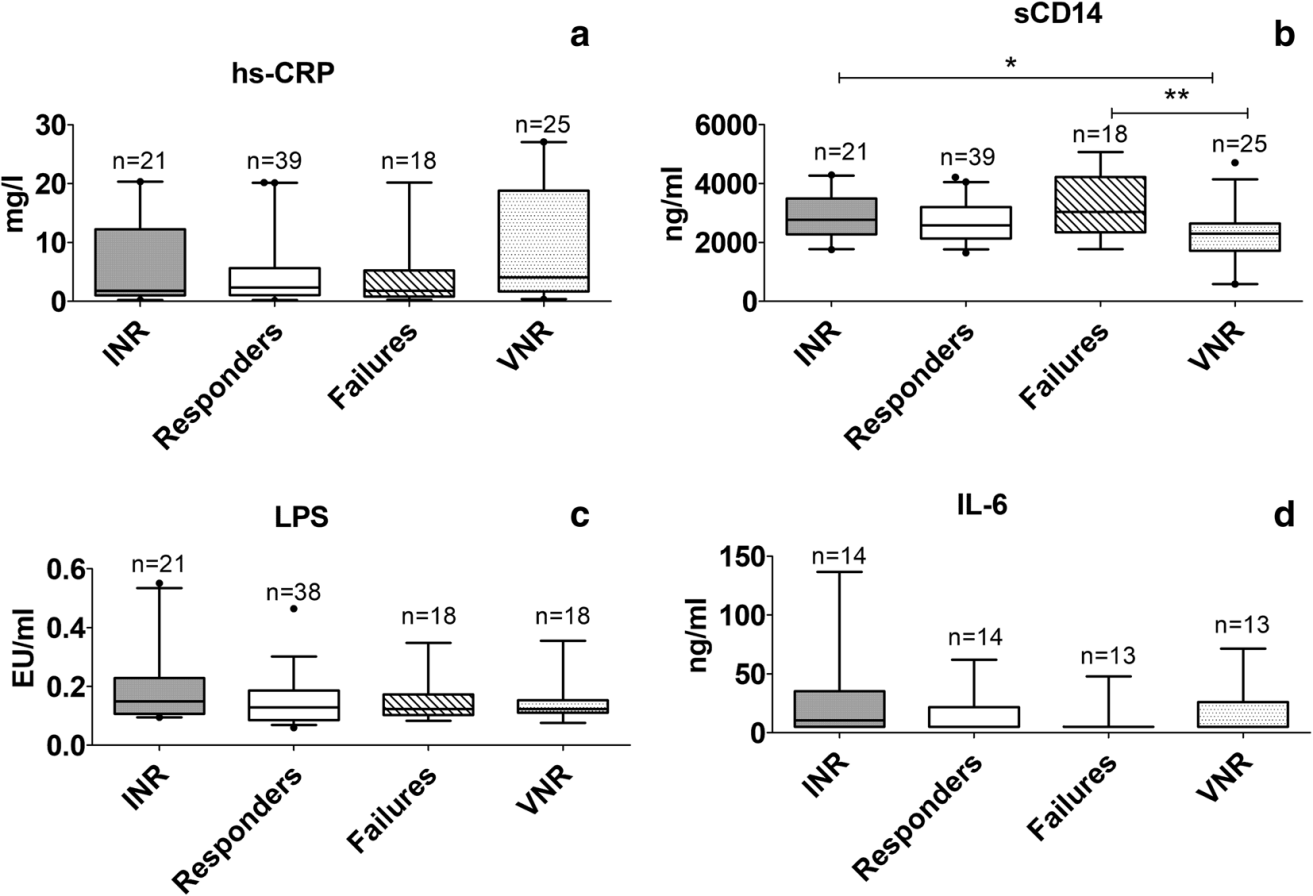
Comparison of immune-activation markers in four groups of the study participants. The figure shows levels of **a** hs-CRP (mg/l), **b** sCD14 (ng/ml), **c** LPS (EU/ml) and **d** IL-6 (ng/ml) plotted on Y axis. Different study groups are shown on X-axis of the graphs. Medians values and interquartile ranges for the groups are plotted as bars and error bars. Number of samples used for the analysis are mentioned above the bars. The groups were compared using Kruskal–Wallis test with Dunn’s post test analysis. Significant differences between the groups are indicated by (*)

The levels were further analysed among all concordant and discordant treatment response groups (Fig. 2). Only sCD14, Galectin-9 and percent CD38+ CD8+ cells showed significant differences by Kruskal–Wallis test (p values 0.0039, < 0.0001, and < 0.0001, respectively). VNR group had lower sCD14 levels than INR and failure patients when compared using Dunn’s post test. hs-CRP, LPS and IL-6 levels did not differ significantly among the study groups.

**Fig. 3:**
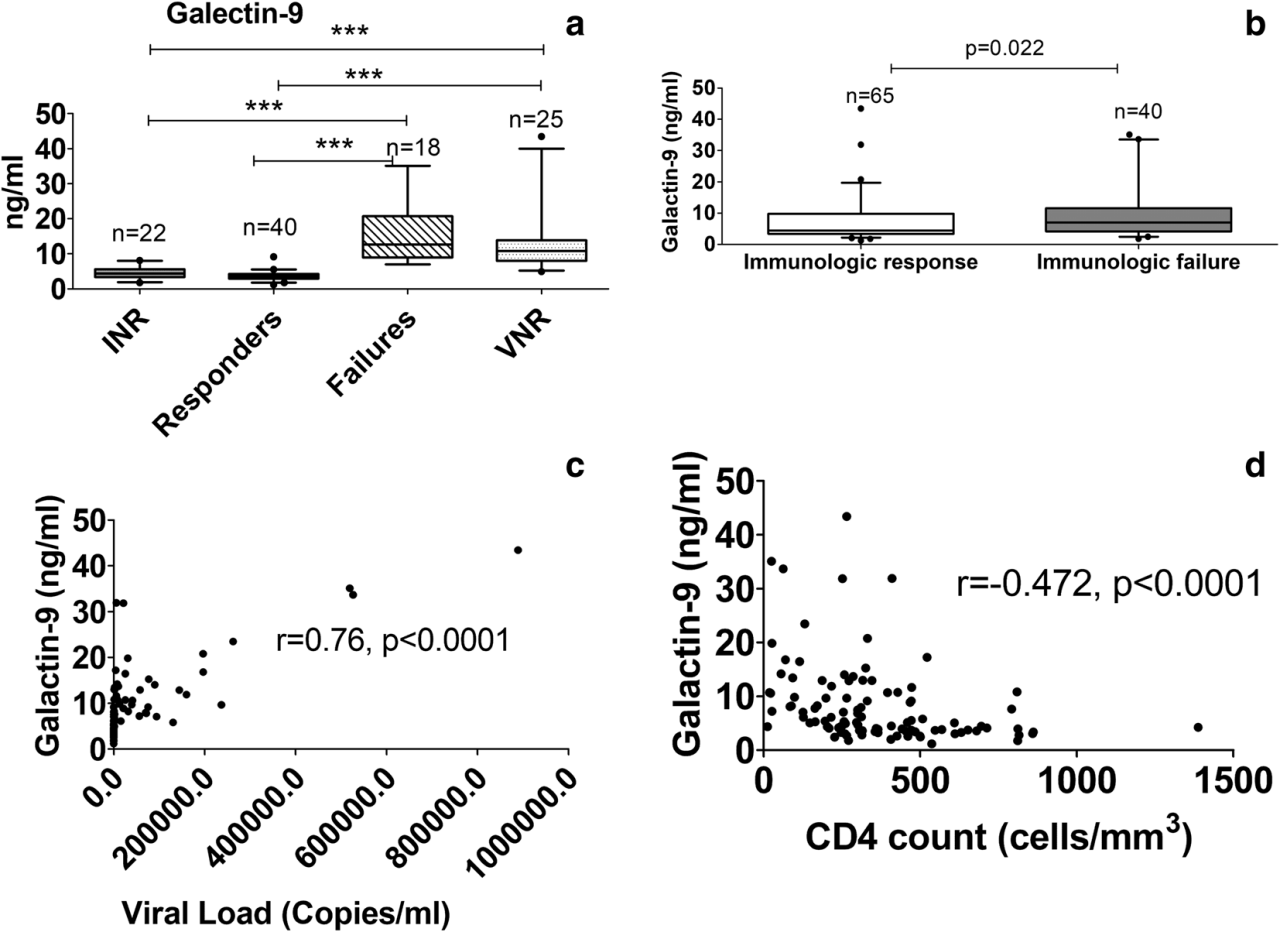
Comparison of Galectin-9 levels in the study groups and their correlation with HIV viral load and CD4 count. **a** Levels of Galectin-9 (ng/ml) plotted on Y axis in different study groups shown on X-axis of the graph. Medians values and interquartile ranges for the groups are plotted as bars and error bars. Number of samples used for the analysis are mentioned above the bars. The groups were compared using Kruskal–Wallis test with Dunn’s post-test analysis. Significant differences between the groups are indicated by (*). **b** Levels of Galectin-9 (ng/ml) plotted on Y axis in participants showing immunologic response versus those not showing immunologic response as shown on X-axis of the graph. P value calculated by Mann–Whitney test is shown in the figure. **c**, **d** Correlation of Galectin-9 levels plotted on Y axis with HIV viral load (**c**) and CD4 counts (**d**) plotted on X axis (n = 103). Spearmen correlation coefficient (r) and p values are also shown in the figure

**Fig. 4:**
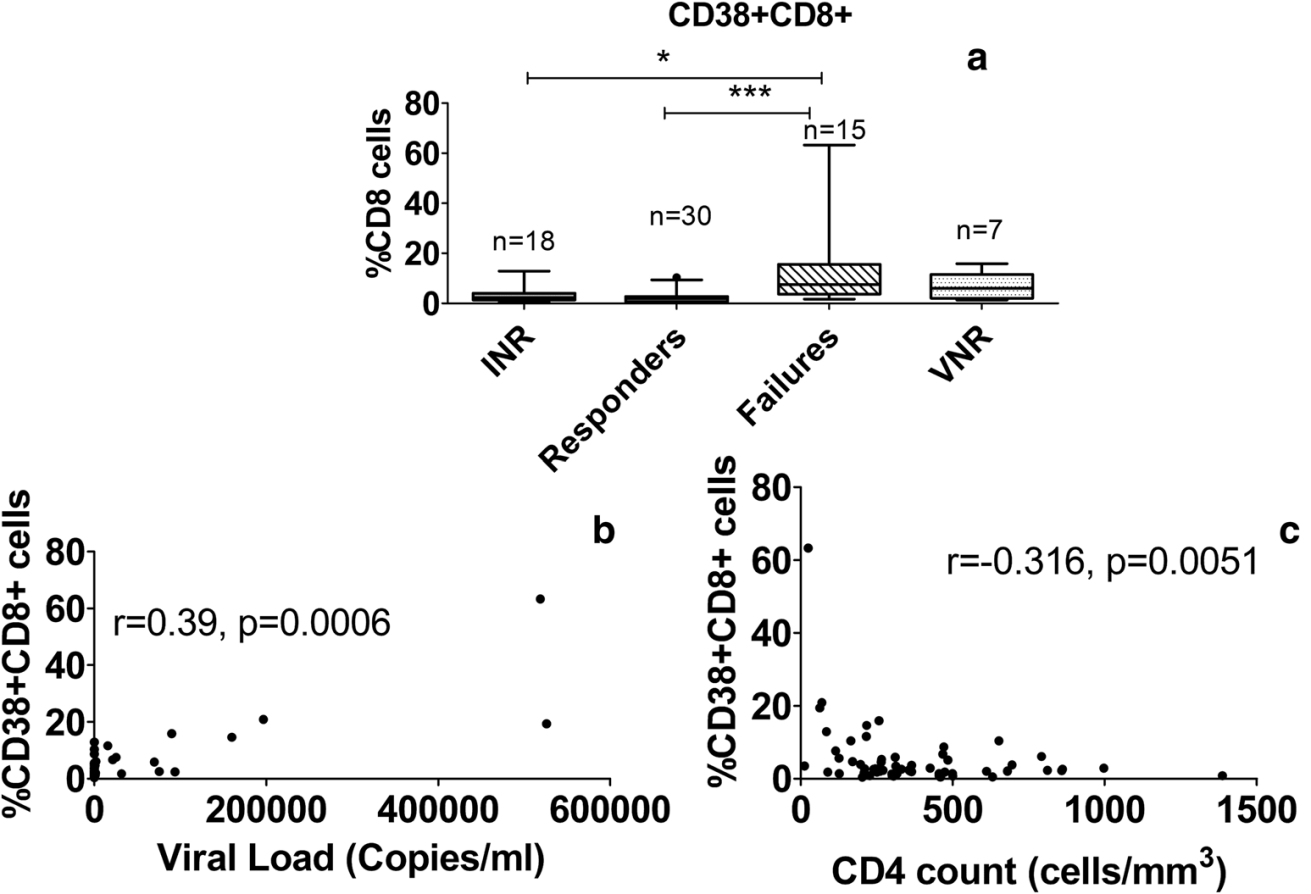
Comparison of frequency of CD38+ CD8+ cells in the study groups and its correlation with HIV viral load. **a** Frequency of CD38+ CD8+ cells plotted on Y axis in different study groups shown on X-axis of the graph. Medians values and interquartile ranges for the groups are plotted as bars and error bars. Number of samples used for the analysis are mentioned above the bars. The groups were compared using Kruskal–Wallis test with Dunn’s post-test analysis. Significant differences between the groups are indicated by (*). **b**, **c** Correlation of frequency of CD38+ CD8+ cells plotted on Y axis with HIV viral load (**b**) and CD4 counts (**c**) plotted on X axis (n = 65). Spearmen correlation coefficient (r) and p values are also shown in the figure

Patients from failures and VNRs groups had significantly higher levels of Galectin-9 in comparison with aviremic groups namely responders and INR groups as shown in Fig. 3a. However, frequency of CD38+ CD8+ cells was significantly higher only in failure group as compared to aviremic patients from INR and responder groups as shown in Fig. 4a. Galectin-9 levels were also found to be significantly lower in patients showing immunologic response (Responders and VNR patients together) in comparison with those without immunologic response (INR and failure patients) as shown in Fig. 3b. Galectin-9 levels correlated positively with plasma viral load values (r = 0.76, p < 0.0001) and negatively with CD4 cell counts (r = − 0.472, p < 0.0001) in these patients (Fig. 3c, d). Similarly %CD38+ CD8+ cells also correlated positively with plasma viral load values (r = 0.39, p = 0.0006) and negatively with CD4 cell counts (r = − 0.316, p = 0.0051) as shown in Fig. 4b, c.

**Fig. 5:**
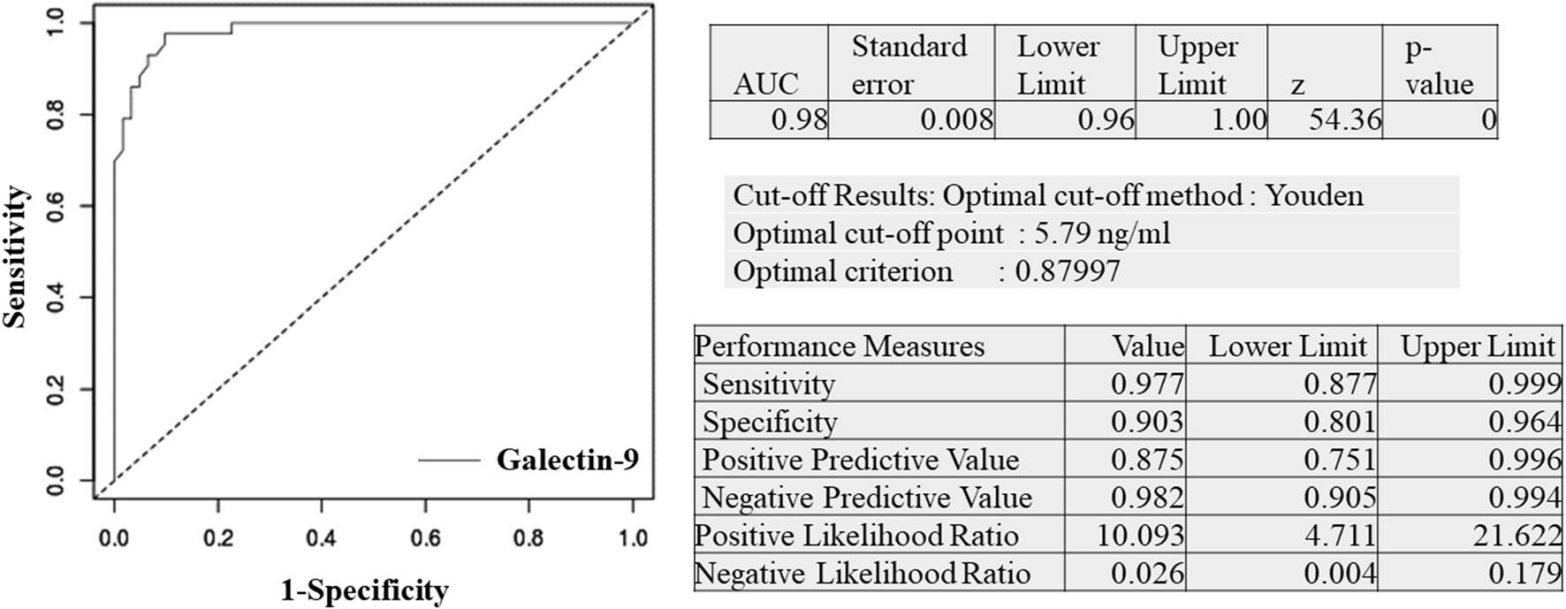
Receiver operating characteristic (ROC) curve for Galectin-9 levels. ROC curve is plotted for Galectin-9 levels for differentiating viremic and aviremic HIV infected patients (n = 103). Sensitivity and 1-specificity are plotted on Y and X axes, respectively. The figure also shows AUC, cut-off point, sensitivity, specificity, predictive values and likelihood ratio for the values as analyzed by easyROC: a web-tool

Since plasma Galectin-9 values correlated strongly with viral load values, ROC analysis was done to determine discriminatory potential of Galectin-9 (Fig. 5). Area under ROC curve (AUC) for Galectin-9 levels was 0.98. Cut off of 5.79 ng/ml was identified to differentiate patients with viremia from those without viremia with sensitivity and specificity of 0.97 and 0.90, respectively.

## Discussion

We screened systemic immune activation markers in HIV infected patients showing immuno-virologic concordant and discordant responses to anti-retroviral therapy for their possible role in identifying HIV infected patients with viremia. Immune activation markers are likely to be influenced by immune status of the patients and may vary depending on the extent of immunosuppression in these patients. Additionally, presence of other co-existing infections may influence their levels. Hence we considered including immune-virologically discordant patients for representing viremic and aviremic individuals with differing degrees of immunosuppression. Moreover, we had also shown that patients with immunologic non-response were likely to have frequent co-existing infections [20] possibly impacting levels of immune activation markers.

Among the systemic immune activation markers studied, only plasma Galectin-9 levels and frequency of CD38 expressing CD8 cells differed significantly between viremic and aviremic patients. These markers were significantly high in viremic groups as compared to the groups with aviremia. Plasma Galectin-9 levels and %CD38+ CD8+ also correlated positively with viral loads in these patients indicating association of these markers with viral replication. CD38 expression has also been shown correlate with viremia and had been proposed as a surrogate biomarker of viremia [9, 10]. Positive correlation between Galectin-9 levels and plasma HIV viral load was detected in a couple of studies [12, 21]. Galectin-9 has been shown to potently reactivate latent HIV in CD4 + T cells ex vivo [12]. Enhanced HIV transcription by Galectin-9 has been shown through T cell receptor (TCR) based ERK signalling [22]. Since viremic patients in our study were virologically failing patients on ART, we did not have viral load values at a very high end of the viral load scale. A larger study with more number of patients having a high level viremia needs to be conducted to confirm the findings. We also found inverse correlation of Galectin-9 levels with CD4 cells counts as reported previously [23] indicating a role of Galectin-9 in HIV disease progression.

Galectin-9 levels were further used for ROC analysis for determining their predictive value in identifying viremic individuals. AUC value of 0.98 indicated high accuracy of these levels in identifying viremic patients. The analysis showed more than 90% sensitivity and specificity in identifying viremic patients at the cut off levels of 5.79 ng/ml. Positive predictive value (87%) for the levels was slightly lower than the negative predictive value (98%). Very high sensitivity and negative predictive value indicated role of the levels as a screening test for identifying viremic patients. However considering its lower positive predictive value and specificity, the results would be required to be confirmed further by viral load test to avoid misidentifying aviremic individuals as those having virologic failure. The rate of virologic failure has been shown to vary from 2.9 to 26.0% in patients in Sub-Saharan Africa and Southeast Asia [24] indicating viral suppression in more than 70% individuals who might not require viral load testing if a screening test is used having a huge financial implication. Lower specificity of these levels is likely because they tend to increase in other infectious diseases also [25–30]. INR patients, who showed higher frequency of infectious diseases in our study [20], had significantly lower Galectin-9 levels than both the viremic groups. Patients from VNR and failure groups are also more likely to suffer from opportunistic infections [31] which might influence Galectin-9 levels. However HIV viral load values have also been shown to increase in presence of coinfections [29] and hence, viral load testing is not recommended within 4 weeks of any diagnosed infection [32].

Other markers like hs-CRP, sCD14, LPS and IL-6 did not vary significantly among viremic and aviremic individuals. Virally suppressive ART had been shown to have no effect on CRP levels in one of the studies [33] and could be a possible reason for similar levels detected in viremic and aviremic individuals in our study. Similarly, no difference in sCD14 and LPS levels has been reported in virally suppressed versus those who are not suppressed [34]. However, the same study reported positive association of IL-6 with HIV viral RNA copies [34]. In contrast, one of the studies had shown that although pre-treatment plasma IL-6 levels correlated weakly with HIV-1 viral load, they failed to decrease proportionately with the viral load after ART [35]. Such conflicting results might be possibly because of multiple factors like the extent of immune reconstitution, presence of co-existing conditions influencing levels of inflammatory markers. Interestingly, sCD14 levels were significantly lower in VNR group than Failure as well as INR groups. Higher sCD14 levels were shown to be associated with immunologic failure [36]. Since VNR patients did not fail immunologically they might not have had higher sCD14 values.

## Conclusion

Thus systemic immune activation markers like hs-CRP, sCD14, IL-6, LPS did not vary significantly in viremic and aviremic individuals precluding their role as surrogate markers of viremia in HIV infected patients on ART. Among the two markers, plasma Galectin-9 levels and frequency of CD38CD8+ cells which differed significantly in these individuals, plasma Galectin-9 levels correlated strongly with viral load values. ROC curve analysis demonstrated very high sensitivity and slightly lower specificity of the levels in diagnosing the patients with viremia suggesting its role as a screening test for identifying viremic HIV infected patients on ART. This cheaper and simpler ELISA test could help to cut down cost of HIV management tremendously and also might help to increase coverage of virologic monitoring even in resource-limited settings and hard to reach population. However, the findings need to be confirmed on a larger sample size and in HIV infected patients with varying durations of ART since the patients included in the study were on ART for 1 year.

## Data Availability

Data supporting the findings are available in the results, in the tables and figures of the manuscript.

## Abbreviations

HIV: Human immunodeficiency virus
ART: Anti-retroviral therapy
VL: HIV viral load
INR: Immunologic non-responders
VNR: Virologic non-responders
hs-CRP: High sensitivity C-reactive protein
sCD14: Soluble CD14
LPS: Bacterial lipopolysaccharide
IL-6: Interleukin-6
ROC: Receiver operating characteristic curve
AUC: Area under ROC curve
